# Recruitment of young adult cancer survivors into a randomized controlled trial of an mHealth physical activity intervention

**DOI:** 10.1186/s13063-022-06148-5

**Published:** 2022-04-04

**Authors:** Carmina G. Valle, Lindsey N. Camp, Molly Diamond, Brooke T. Nezami, Jessica Gokee LaRose, Bernardine M. Pinto, Deborah F. Tate

**Affiliations:** 1 Department of Nutrition, Gillings School of Global Public Health and School of Medicine, University of North Carolina at Chapel Hill, Chapel Hill, NC USA; 2 Lineberger Comprehensive Cancer Center, University of North Carolina at Chapel Hill, Chapel Hill, NC USA; 3 Department of Health Behavior, Gillings School of Global Public Health, University of North Carolina at Chapel Hill, Chapel Hill, NC USA; 4 Present address: William F. Connell School of Nursing, Boston College, Chestnut Hill, MA USA; 5 Department of Health Behavior and Policy, School of Medicine, Virginia Commonwealth University, Richmond, VA USA; 6 College of Nursing, University of South Carolina, Columbia, SC USA

**Keywords:** Young adults, Cancer survivors, Physical activity, Recruitment methods, Social media, Cost

## Abstract

**Purpose:**

Few studies have recruited young adult cancer survivors (YACS) from around the USA into remotely-delivered behavioral clinical trials. This study describes recruitment strategies used in the IMproving Physical Activity after Cancer Treatment (IMPACT) study, a 12-month randomized controlled trial of a mobile physical activity intervention for YACS.

**Methods:**

We conducted formative work to guide development of recruitment messages and used a variety of methods and channels to recruit posttreatment YACS (diagnosed ages 18–39, participating in < 150 min/week of moderate-to-vigorous intensity activity). We used targeted social media advertisements, direct mailings, clinical referrals, and phone calls to potentially eligible individuals identified through local tumor registries. We also asked community organizations to share study information and advertized at a national conference for YACS.

**Results:**

The final sample of 280 participants (23% identified as racial/ethnic minority individuals, 18% male, mean 33.4 ± 4.8 years) was recruited over a 14-month period. About 38% of those who completed initial screening online (*n* = 684) or via telephone (*n* = 63) were randomized. The top recruitment approach was unpaid social media, primarily via Facebook posts by organizations/friends (45%), while direct mail yielded 40.7% of participants. Other social media (paid advertisements, Twitter), email, clinic referrals, and conference advertisements each yielded 3% or fewer participants. The most cost-effective methods per participant recruited were unpaid social media posts and direct mailings.

**Conclusions:**

The IMPACT trial successfully met enrollment goals using a national strategy to recruit physically inactive YACS. Our approaches can inform recruitment planning for other remotely-delivered intervention trials enrolling YACS.

**Trial registration:**

ClinicalTrials.govNCT03569605. Registered on 26 June 2018.

**Supplementary Information:**

The online version contains supplementary material available at 10.1186/s13063-022-06148-5.

## Background

There are ~ 630,660 young adult cancer survivors, ages 20–39, in the USA [1]. Young adult cancer survivors (YACS), diagnosed between the ages of 18 and 39, are a vulnerable and underserved group of survivors that are at risk for several chronic conditions, along with other long-term and late effects related to cancer and its treatment [2, 3]. The cumulative risk for chronic health conditions increases with age in YACS [4]. Further, a cancer diagnosis during young adulthood can interrupt an already challenging development period, marked by major life transitions with respect to education, employment, finances, physical, and psychosocial development [5–7]. This can result in an array of unique needs for information and support as young adults progress from cancer treatment into survivorship [8–10]. With more life-years affected by cancer relative to other age groups [11], YACS may be faced with more decades at risk for chronic conditions and poor health outcomes [12]. Thus, there is a strong need for research focused on meeting the age-specific needs of YACS and reducing their morbidity and disease risks [3, 13, 14].

Despite calls for increased attention and research among this underserved subgroup of cancer patients and survivors [3], researchers have been challenged to recruit YACS to participate in clinical research studies [15]. Enrollment of adolescents and young adults with cancer (AYAs) into clinical trials ranges from 6% to 18% [16, 17], with lower rates of enrollment among those 20–39 years old [16, 18]. While systematic reviews have identified barriers and facilitators to clinical trial enrollment among AYAs with cancer, these have focused on treatment-related trials [19, 20], and limited research is available to guide enrollment into behavioral clinical trials for cancer survivors. Previous studies have identified approaches for recruiting cancer survivors into observational and behavioral research studies using social media [21–23], population-based survey methods [24, 25], and direct mail to individuals identified through state cancer registries [24–26]. Among the studies describing the recruitment of YACS, social media [22, 27], and direct mailings [24, 25, 28] have produced higher recruitment yields, but few have focused on recruitment into behavioral clinical trials [28]. Further, while formative work is recommended to inform recruitment strategies [29], there is limited reporting of formative research findings used to recruit YACS into behavioral clinical trials, and there is a need for studies to report the cost-effectiveness of recruitment strategies [30]. To our knowledge, costs by recruitment methods have yet to be described in the context of recruiting YACS into behavioral intervention trials.

Overall, limited research exists to guide approaches to both efficiently and cost-effectively recruit YACS into behavioral clinical trials. Identifying effective recruitment strategies can inform future efforts to engage YACS in participating in clinical trials more broadly. Thus, the objective of this paper is to describe the recruitment messages and strategies, costs, and yield of recruitment methods for the IMproving Physical Activity after Cancer Treatment (IMPACT) study, a 12-month randomized controlled trial of a theory-based, mobile physical activity intervention designed specifically for YACS. The design and protocol of the IMPACT trial is published elsewhere [31]. This paper describes (1) the plan and messages used to recruit YACS into this trial, (2) recruitment strategies used and their yield, and (3) costs associated with recruitment strategies.

## Methods

### Participants

This project was a single-site clinical trial, implemented at the University of North Carolina at Chapel Hill (UNC). All study procedures were reviewed and approved by the UNC Lineberger Protocol Review Committee and Institutional Review Board (IRB # 16-3409). All study participants provided online informed consent to participate. The IMPACT study goal was to recruit and enroll 280 YACS who were currently age 18–39, were diagnosed with cancer between ages 18–39 and within the last 10 years, had completed active therapy, could read, write, and speak English, participated in less than 150 min/week of moderate-to-vigorous intensity activity, and had Internet access, a mobile phone, and a text messaging plan. YACS were recruited for a formative research phase (*n* = 10; January–February 2018) and for the randomized trial (*n* = 280; August 2018–October 2019).

### Formative research and message development

**Fig. 1:**
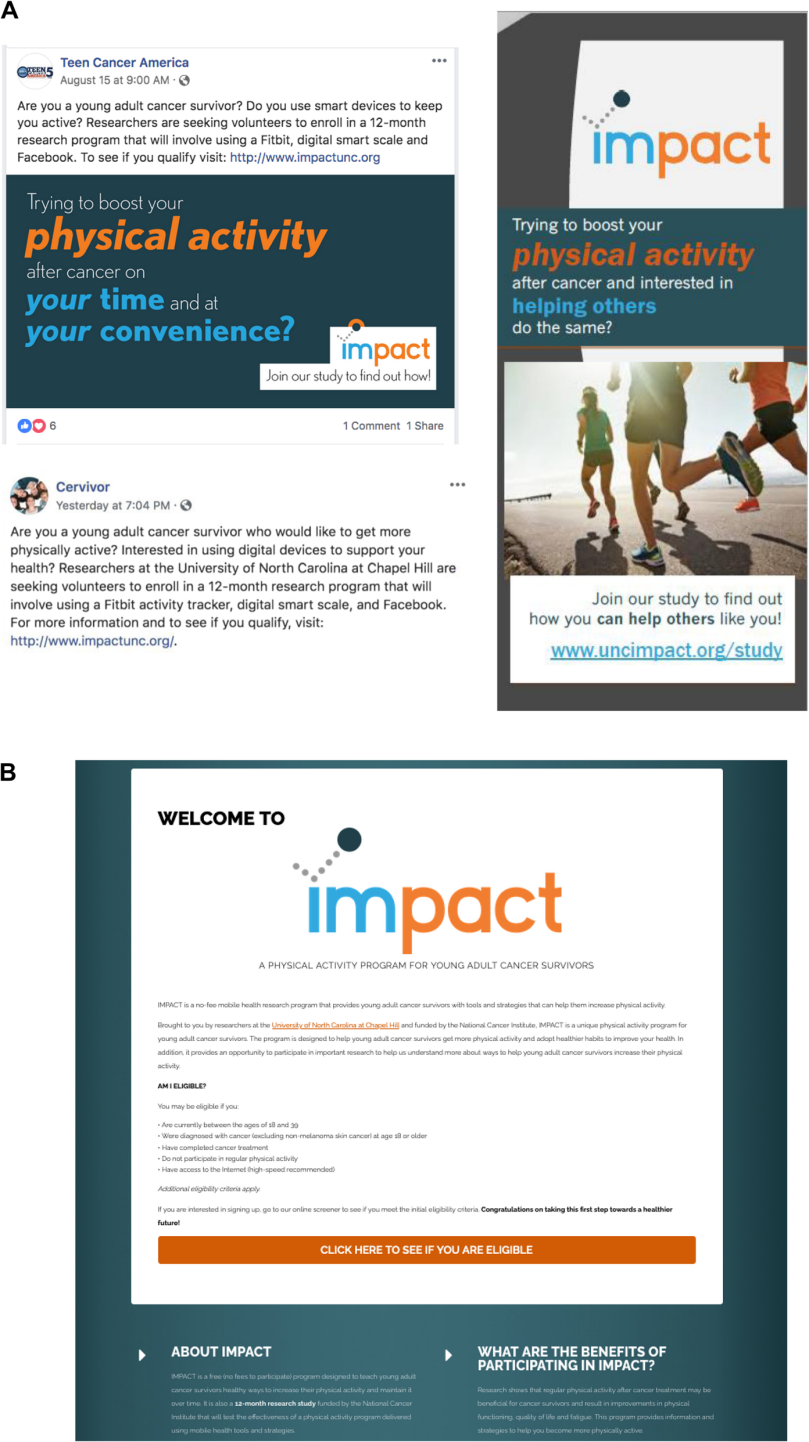
IMPACT study recruitment materials. **A** Recruitment messages, including example Facebook posts by cancer organizations and study brochure. **B** Recruitment website

In preparation for the randomized trial, we conducted a 6-week pilot of study procedures among 10 YACS. Participants were assessed at baseline, 3, and 6 weeks with similar measures used in the main trial at baseline, 3, and 6 months [31]. The 3-week online questionnaire asked participants for feedback on four potential recruitment messages that emphasized YACS’ motivations for participating in research and challenges with recruiting YACS to clinical trials, as documented in previous research [32]. Four different recruitment messages focused on getting back to pre-cancer fitness or physical activity levels, struggling with fitness or physical activity after cancer, interest in helping others, or getting active on their own time and at their convenience (Additional file 1 shows recruitment messages). Participants were presented with six questions. Each question paired two of the four recruitment messages and asked participants to choose which message makes them most want to join a study. An open-ended question asked about their preferences and what they liked or did not like about messages. Among the four messages, the most highly endorsed was: “Are you struggling with physical activity after cancer and interested in helping others with the same problem? Join our study to find out how you can help others like you!” Many participants noted that they did not like the words *fitness* and *pre-cancer.* Participants highlighted *physical activity*, *helping others*, and *on your time and at your convenience* as preferred language for recruitment. Based on this formative work, we finalized messages (Fig. 1), developed a recruitment timeline and plan, and initiated recruitment.

### Recruitment plan

**Fig. 2:**
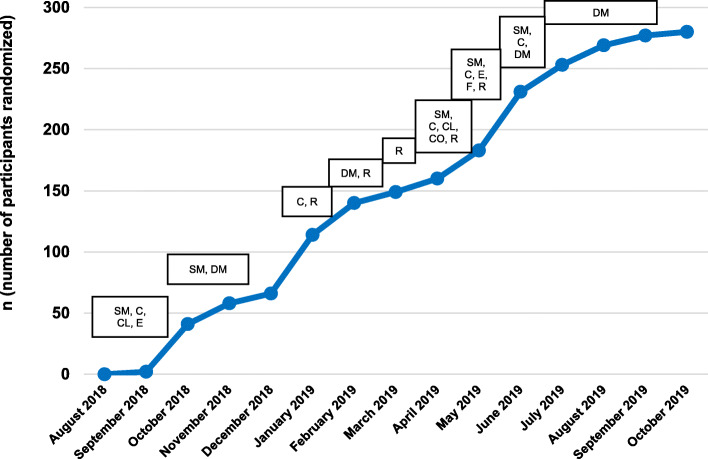
Recruitment strategies from August 2018 to October 2019. SM, social media posting; C, community organizations contacted; CL, clinic contact; CO, conference; E, emails to listserv or smaller groups; F, online forum posting; DM, direct mailing; R, registry. Not shown: flyers posted or “word of mouth”

We planned to use a variety of recruitment channels that had been successful in our previous studies, including social media and reaching out to cancer organizations to share study information, and to maximize potential to reach YACS around the USA [33]. Considering our goal of recruiting at least 25% of participants from racial/ethnic minority groups since YACS in these groups are disproportionately affected by cancer [14], we planned to use direct mail to reach potentially eligible individuals who received care from the UNC Health system, a public system serving all North Carolina residents. We also made concerted efforts to share information with community organizations focused on diverse groups of YACS. We planned to recruit in cohorts of at least 5 individuals, with the goal of randomizing a new cohort every 2 or 4 weeks until reaching the desired sample size of 280. We advertised continuously from August 2018 through early September 2019 (Fig. 2) and tracked enrollment yield to inform prioritization of approaches over time.

### Recruitment messages

Our final recruitment messages included language informed by our formative work. We worked with the Connected Health Applications and Interventions (CHAI) Core, an NIH-funded shared resource, to develop study graphics and fonts that we used consistently across advertisements and a mobile responsive recruitment website (Fig. 1). We used two general recruitment messages in graphics, postcards, and brochures with the goal of attracting a wide range of potentially interested YACS. Each advertisement was distributed via different recruitment channels (Facebook, Instagram, direct mailings, other strategies) and included unique URLs to distinct website landing pages with identical study information. All advertisements encouraged potential participants to visit a recruitment website with information on the study purpose, eligibility criteria, benefits of participating (e.g., activity tracker, wireless scale), program details, and a link to a preliminary online screener in a secure REDCap survey [34]. The REDCap survey tracked the originating URL through which each individual accessed the screener, which enabled identification of the distinct message and channel that directed individuals to the recruitment website. Additionally, a question in the online screener asked individuals how they heard about the program (see the “Measures” section for details).

Study staff contacted individuals who were initially eligible after online screening to complete additional telephone screening. Once an individual was determined to be eligible, s/he received an emailed link to complete an online informed consent. Following consent, individuals were asked to complete baseline questionnaires and wear an ActiGraph accelerometer to record baseline physical activity levels for 7 days. Individuals were eligible for randomization only if all baseline measurements were complete and the accelerometer was returned with adequate wear time (i.e., wear > 10 h on ≥ 4 days).

### Recruitment strategies

#### Social media

Recruitment advertisements on social media primarily used two platforms (i.e., Facebook, Instagram) with unpaid and paid approaches. As in our previous work [33], we focused initially on unpaid advertising. We reached out to previously established contacts at local and national community-based organizations and cancer centers and asked them to post recruitment information using their preferred approaches. With permission, we also posted recruitment information directly in organizational Facebook groups. Additionally, we conducted online searches for other organizations dedicated to young adults with cancer, contacted them, provided them with IRB-approved recruitment ads for their consideration, and asked if they would be willing to share study information with their constituents (see Additional file 2 for organizations that shared study information). For paid advertising/sponsored posts, we launched Facebook/Instagram campaigns.

#### Direct mailings

We obtained a list of potentially eligible participants from the UNC Carolina Data Warehouse for Health, a central data repository with clinical and administrative data from the UNC Health system, including patient demographics and diagnoses. Potentially eligible survivors were sent an approach letter from the study Principal Investigator, inviting them to participate and visit the recruitment website or call study staff to be screened. We mailed a total of 3458 letters over a year-long period that included either a postcard or brochure directing interested individuals to the recruitment website.

#### Email

When contacting local and community-based cancer organizations to inquire about their willingness to distribute study information, we offered them IRB-approved language for distribution via email listservs and contacts. Emails were sent to and distributed by cancer organizations, program managers, and clinicians of adolescent and young adult cancer programs.

#### UNC Health Registry/Cancer Survivorship Cohort

The UNC Health Registry/Cancer Survivorship Cohort is a cohort of over 7500 cancer patients enrolled from UNC oncology clinics [35]. Registry staff identified potentially eligible individuals based on medical record data and study eligibility criteria, and study staff removed individuals who had already received letters and completed screening (*n *= 11). Between January and May 2019, registry staff attempted to contact 101 individuals by telephone to gauge study interest and conduct an initial eligibility screening. Then, study staff followed up with interested and initially eligible individuals to complete screening by telephone.

#### Other

We produced flyers and brochures to advertise the study at medical clinics, community events, and cancer conferences. Additionally, we actively recruited individuals at a cancer conference dedicated to young adults (i.e., CancerCon, April 2019), where we had an exhibitor table with information displays, flyers, and computer tablets to facilitate online screener completion. Two study staff were available to discuss the study with potentially interested individuals.

### Measures

#### Recruitment channels

A question in the online screener asked participants to check all recruitment channels through which they heard about the study (e.g., Facebook post by friend, family, or co-worker; email from cancer organization; letter). The URL landing page through which each individual accessed the preliminary screener was also collected in the REDCap online screener. The URLs signaled the method through which participants were exposed to the study advertisement (i.e., Facebook, Instagram, direct mailing, or another method). Primary recruitment method was determined by the URL landing page. For individuals with missing or indistinguishable URLs (*n *= 18), the participant’s reported recruitment channel was designated as a primary recruitment channel.

#### Recruitment metrics

We collected data on the numbers of individuals screened and enrolled via each recruitment strategy. We tracked the number of individuals approached using direct mail and the registry. Participation rate was calculated as the number of individuals who enrolled and participated divided by the number of fully eligible individuals. For each recruitment channel, we calculated recruitment yield as the number of participants enrolled divided by the total final sample (*n* = 280).

### Statistical Analyses

We provide descriptive statistics, including means and standard deviations for continuous variables and counts and frequencies for categorical variables, by demographic variables and recruitment channel. Additionally, we conducted logistic regression analyses to compare yields by demographic and recruitment channel subgroups using SPSS Statistics (Version 27). Yield and estimated cost per participant recruited (*n* = 280) by recruitment channel are provided.

## Results

**Fig. 3:**
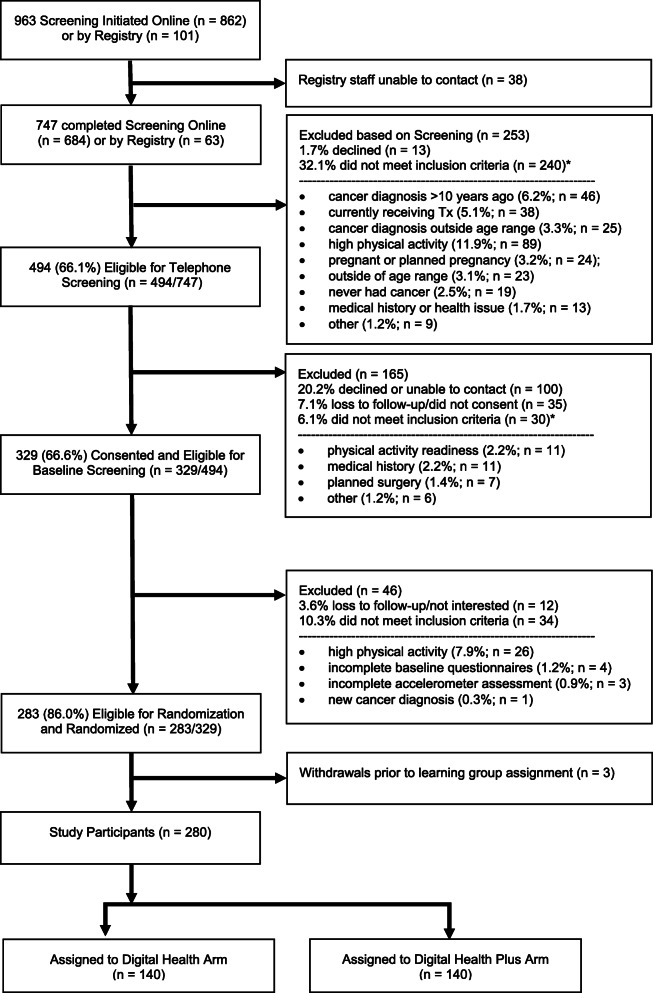
CONSORT diagram. *Asterisk indicates there may be more than 1 reason for ineligibility

The flow of participant enrollment, including the number of individuals screened, consented, and randomized and reasons for ineligibility, is shown in the CONSORT diagram (Fig. 3). Of 747 individuals who completed initial screening criteria related to age, cancer history, activity levels, pregnancy status, current participation in a physical activity/weight loss program, and technology access, 66.1% were initially eligible. The most common reasons for an ineligibility after initial screening were high activity level (37.1%) and a cancer diagnosis over 10 years ago (19.2%).

Of individuals that were eligible after initial screening, 79.8% (*n* = 394) completed phone screening, 73.7% were eligible (*n *= 364), and 66.6% consented (*n* = 329). Among those who did not meet inclusion criteria based on phone screening (*n* = 30), the most common reasons for exclusion were physical activity readiness (i.e., medical conditions that could limit exercise) (36.7%), medical history (36.7%), and planned surgery (23.3%).

Of those who were eligible after complete screening, 90.4% completed the online consent process and were eligible for baseline assessment. About 14% (*n* = 46) of those who consented became ineligible for randomization, with 12 no longer interested in participating and 34 not meeting inclusion criteria. The most common reason for ineligibility for randomization was high activity level, as determined by accelerometer assessment in 26 individuals.

After completion of baseline assessments, a total of 283 individuals met eligibility criteria for randomization or 37.9% of 747 who completed initial screening. Subsequently, three individuals were withdrawn prior to receiving their group assignment. Two became ineligible for medical reasons, and one did not complete the synchronous kickoff video session with study staff, yielding a final study sample of 280 participants (85.1% participation rate among individuals eligible following complete screening; *n* = 280 of 329). Characteristics of the final study sample are described in Table 1 by recruitment channel. On average, participants were 33.4 (4.8) years old and 3.7 (2.4) years post diagnosis. Eighteen percent of the sample identified as male, 22.9% identified as individuals of color, and 92.5% had completed some college or more education. The four most common cancers were breast, Hodgkin’s lymphoma, melanoma, and thyroid cancers.

**Table 1:** Baseline demographic characteristics of 280 study participants: overall and by recruitment channel

	All randomized *n* = 280 *n* (%)	Social media *n* = 136 *n* (%)	Direct mailing *n* = 114 *n* (%)	Other *n* = 30 *n* (%)
Age (years)				
18–25	26 (9.3%)	13 (9.6%)	12 (10.5%)	1 (3.3%)
26–35	157 (56.1%)	77 (56.6%)	63 (55.3%)	17 (56.7%)
36–39	97 (34.6%)	46 (33.8%)	39 (34.2%)	12 (40.0%)
Sex				
Male	51 (18.2%)	9 (6.6%)	37 (32.5%)	5 (167%)
Female	229 (81.8%)	127 (93.4%)	77 (67.5%)	25 (83.3%)
Race*				
Asian	5 (1.8%)	3 (2.2%)	1 (0.9%)	1 (3.3%)
Black	32 (11.5%)	11 (8.1%)	17 (14.9%)	4 (13.3%)
White (non-Hispanic)	215 (77.1%)	108 (79.4%)	85 (74.6%)	22 (73.3%)
Multiple races	10 (3.6%)	5 (3.7%)	4 (3.5%)	1 (3.3%)
Other	17 (6.1%)	8 (5.9%)	7 (6.1%)	2 (6.7%)
Ethnicity				
Hispanic	23 (8.2%)	11 (8.1%)	10 (8.8%)	2 (6.7%)
Education				
≤ High school graduate	21 (7.5%)	8 (5.9%)	13 (11.4%)	0 (0)
Any college	160 (57.1%)	73 (53.7%)	68 (59.6%)	19 (63.3%)
Post college	99 (35.4%)	55 (40.4%)	33 (28.9%)	11 (36.7%)
Time since cancer diagnosis (years)				
< 5 years	205 (73.2%)	97 (71.3%)	87 (76.3%)	21 (70%)
≥ 5 years	75 (26.8%)	39 (28.7%)	27 (23.7%)	9 (30%)
Cancer type^#^				
Breast	63 (22.5%)	43 (31.6%)	15 (13.2%)	5 (16.7%)
Hodgkin lymphoma	31 (11.1%)	21 (15.4%)	8 (7.0%)	2 (6.7%)
Melanoma	27 (9.6%)	5 (3.7%)	16 (14.0%)	6 (20.0%)
Thyroid	30 (10.7%)	11 (8.1%)	17 (14.9%)	2 (6.7%)
Children at home				
Yes	132 (47.1%)	58 (42.6%)	60 (52.6%)	14 (47.1%)
No	148 (52.9%)	78 (57.4%)	54 (47.4%)	16 (53.3%)

Note: * Missing race *n *= 1; ^#^Four most common cancers

The final sample of 280 participants was recruited from August 2018 to October 2019 and randomized at a rate of 20 participants/month (Fig. 2). The top recruitment approach was social media, with nearly half of randomized participants in the final sample (48.6%, 136 of 280) recruited via social media posts while direct mail yielded 40.7% of participants. Among randomized participants recruited through social media, 92.6% were recruited through Facebook posts by organizations/friends (45% of final sample), 5.9% from paid advertisements, and 1.5% through Twitter posts. Other recruitment channels (i.e., email, list servs, clinic referrals, and conference advertisements) each yielded 3.6% or fewer study participants.

### Recruitment methods for subgroups

**Fig. 4:**
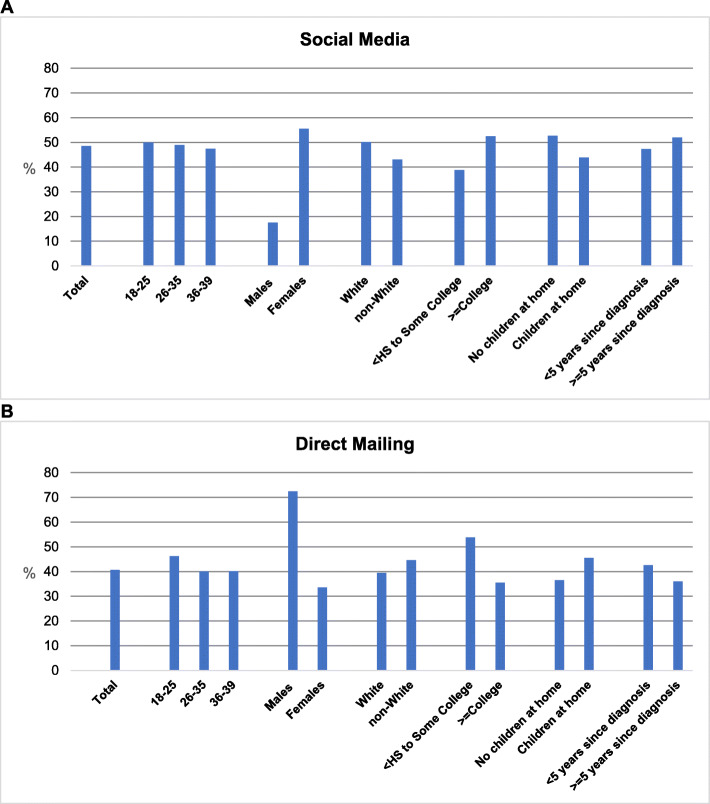
Recruitment of subgroups by **A** social media and **B** direct mailing. Percentages are within subgroup (e.g., within male/female, percent of sample recruited via direct mailing; among males, 72.5% recruited via direct mailing and 27.5% not recruited via direct mailing; among females, 33.6% recruited via direct mailing and 66.4% not recruited via direct mailing)

Subgroups of participants were more likely to be recruited by social media or direct mail. Figure 4 shows the top two recruitment approaches, social media, and direct mail, and the corresponding sample categorized by demographic characteristics at baseline. Among those recruited through social media, greater proportions of female compared to male participants were recruited [OR (95%CI): 5.81 (2.70, 12.50); *p<*.0001]. Additionally, social media was more effective for recruiting participants with college degrees compared to those with less education [OR (95% CI): 1.75 (1.03, 2.96); *p*=.039]. Among men enrolled, 72.5% (37 of 51) were recruited by direct mail, versus 33.6% of women (77 of 229). Direct mailings were more likely to recruit male than female participants [OR (95% CI): 5.22 (2.66, 10.23); *p*<.0001] and participants with less than a college degree compared to those who were at least college graduates [OR (95% CI): 2.11 (1.25, 3.57); *p*=.005]. Among those with less than a college degree, 53.8% were recruited by direct mail (43 of 80), and 38.8% were recruited via social media, while among those with a college degree 35.5% were recruited through direct mail (71 of 200) and 52.5% were recruited via social media.

The estimated costs per recruitment channel and per participant recruited are outlined in Table 2. Personnel costs specific to recruitment activities are not included (e.g., time spent contacting organizations or posting to social media) as these were not documented by method and could not be isolated from other activities undertaken by study staff. The most costly methods were the Health Registry and conference/community events, which cost approximately $516 and $386 per participant randomized. The most affordable paid recruitment method that had the highest yield was direct mailings, at a cost of $35 per individual recruited. Notably, recruitment methods that did not incur payments beyond personnel costs, largely social media posts initiated by the research team and through contacts to cancer organizations, yielded half of the final sample of randomized participants.

**Table 2:** Recruitment channels, yields, and associated costs

Recruitment channel	Total cost^a^	Participants recruited with method *n* (%)	Cost per participant recruited
Health Registry/Cancer Survivorship Cohort	$4125.00	8 (2.9%)	$515.63
Community events and conferences (includes marketing products, brochures, exhibitor registration)	$3857.71	10 (3.6%)	$385.77
Social media paid advertising	$1610.37	8 (2.9%)	$201.30
Direct mailing (mailing list, printing, postage)	$4007.01	114 (40.7%)	$35.15
Social media posts directing to recruitment website	N/A	128 (45.7%)	N/A
Study referral (via clinic, listserv, word of mouth, other participants)	N/A	8 (2.9%)	N/A
Email (from friend or organization)	N/A	4 (1.4%)	N/A
Total^b^	$13,725.09	280 (100%)	$49.02

Note: ^a^Personnel costs not included. ^b^Includes cost for recruitment website domain of $125

## Discussion

The IMPACT trial successfully recruited and randomized 280 YACS from around the USA at a rate of about 20 participants per month. Our efforts were guided by formative work that identified recruitment messages that would resonate with YACS specifically. We used a variety of recruitment strategies and outlets, as well as a mix of online screening followed by telephone to determine eligibility. Twenty-nine percent of individuals that initiated screening and 85% of those that were eligible following complete screening were randomized and included in the final study sample. The highest yield recruitment approach was social media followed by direct mail. Among the final sample recruited via social media, the large majority were through Facebook posts by cancer organizations, friends, or family, highlighting the value of recruitment information shared by community organizations and trusted sources. Importantly, we found that social media approaches were more successful for recruiting women than men and individuals with a college degree, whereas direct mail yielded a higher proportion of men than women and those without a college degree. Overall, our findings could provide useful guidance for recruiting sedentary YACS and for remotely-delivered behavioral intervention trials.

The prior studies that have described recruitment yields of adolescent and young adult cancer survivors (AYAs) have focused on survey studies and reported variable participation rates among individuals identified as eligible. In the AYA HOPE study, 43% of eligible AYAs participated in the survey study [25] and participation rates were 85–86% among AYA female survivors across two national survey studies [22]. Similarly, while enrolling participants into a 12-month randomized trial, we observed a participation rate of 85% among individuals eligible after online and telephone screening. This is higher than previous randomized trials of remotely-delivered physical activity interventions among YACS reporting initial eligibility, which were all pilot trials of short duration that reported participation rates ranging from 59% to 88% [33, 36–38]. Information on recruitment yield based on overall numbers of individuals approached or individuals that initiated screening is less readily available from randomized trials with YACS. Previous yields in physical activity intervention trials among YACS have ranged from 11 to 40% [28, 33, 39], and in the current study, 29% of those who initiated screening were retained in the final sample. A continued challenge when characterizing recruitment yields by various channels is the lack of data on the total number of individuals approached. While this was known for our direct mail and health registry approaches, we were limited in our ability to track the number of individuals approached through other means, such as social media posts and emails by cancer organizations.

Consistent with prior studies that conducted national recruitment of AYAs, we found that social media was the highest yield approach. While recruiting female AYAs into two national survey studies of reproductive health issues, social media and internet approaches had the highest yield of participants; in one study, 60% of participants were recruited via social media, largely through the Stupid Cancer’s Facebook page [22]. In a preliminary report of recruitment of female AYAs into a mixed-methods study of fertility, 37% of individuals who initiated contact with Facebook or Instagram ads posted by cancer organizations enrolled in the study and comprised 72% of the sample (*n*=75) [27]. In the current study, using a similar approach yielded 45% of the final study sample. As in our previous trial [33], we approached several organizations and provided IRB-approved recruitment messages that could be posted on social media sites or emailed to constituents to facilitate sharing information and ease burden on organizations. We observed previously reported advantages of this unpaid social media approach, including reach, peer-to-peer communication, and lower costs [22, 27, 30]. Facebook posts, by community organizations in particular, yielded the highest proportion of participants and enabled us to extend the reach of our advertisements, allowing for them to be communicated by trusted organizations or information sources that individuals may have actively sought out and decided to follow. Our paid social media advertisements were less effective, yielding only 3% of the final study sample at a much higher cost of $201 per individual recruited.

Direct mailings to individuals identified through the local tumor registry yielded the second highest proportion of participants in the final study sample. In a study describing recruitment of 12 YACS into a pilot trial of a physical activity intervention, direct mail to survivors in a hospital-based tumor registry was the most effective strategy yielding 67% of the recruited sample [28]. Other studies have found mailings to be a productive recruitment strategy, including survey studies among AYAs [24, 25] and exercise intervention trials among breast cancer survivors [40–43]. In our exploratory analyses, direct mail yielded the majority of men in the sample and those with lower educational attainment. There is evidence that recruiting underrepresented and/or hardly reached populations through direct mailings to survivors identified through state cancer registries is feasible [26, 42]. In later rounds of our recruitment mailings, we made concerted efforts to direct them to individuals identified as men and/or Black, Asian, American Indian, or Hispanic. Twenty-three percent of our sample identified as a person of color, which is higher than most previous physical activity intervention trials among YACS (range: 6–26%) [33, 36–39], and male participants comprised 18% of the sample, which falls within the ranges reported in previous trials (9–44%) [33, 36–39, 44]. In recruiting a population-based sample for the AYA HOPE study, likelihood of participation was lower in males versus females and non-Hispanic Blacks and Hispanics compared to non-Hispanic Whites [25]. Among our final sample, less than 10% of participants were emerging adults 18–25 years of age. Despite emerging adulthood being recognized as a critical period to promote healthy lifestyle behaviors [45], few studies have reported recruitment yields among emerging YACS enrollees and the mean age of participants in previous intervention trials among YACS is in the early 30s [33, 36, 37, 44]. Additional strategies to enhance participation by YACS that are men, of younger ages, and from diverse racial/ethnic backgrounds are needed. Researchers might consider engaging stakeholders, including men and survivors from racially and ethnically diverse communities, in a community advisory board to further guide the expansion of recruitment approaches, messaging, and program content to enroll YACS who are underrepresented in behavioral clinical trials.

The success of recruitment through social media and direct mail may have been facilitated by several mechanisms. First, our formative work informed the development of recruitment messages that may have been more appealing and relevant to YACS’ motivations to enroll in a behavioral clinical trial, thus attracting interested individuals and improving our response rates [29]. Second, the study design, including remote provision of active intervention components and digital tools to support physical activity in both randomized groups, may have contributed to successful recruitment. Social media and mobile device use is highest among young adults [46, 47], and YACS desire digital and remotely-delivered interventions [48–50]. The remote delivery may have facilitated participation by eliminating the need to travel, a common barrier to participation in cancer clinical trials [19]. Third, the potential benefits and incentives for participation (i.e., activity tracker, wireless scale, payments for completion of assessments) may have increased motivation for YACS to enroll in the study. The recruitment website outlined the potential benefits of participating, and we made concerted efforts via the website, telephone screening, and consent process to ensure that individuals had a clear understanding of what study participation entailed and the importance of completing assessments. Fourth, previous literature has reported that altruism may predict willingness to participate in research [21], and that sense of altruism is prevalent among YACS [51]; some of our recruitment messages attempted to appeal to this and increase motivation for participation. Finally, the use of social media facilitated our timely recruitment, which is consistent with findings of a systematic review on using Facebook to recruit participants into health research [52]. Indeed, a survey of childhood cancer survivors indicated that 79% had positive attitudes about using social media sites for cancer research recruitment [53]. Facebook posts by organizations, friends, or family was the most cost-effective method with the highest yield.

Across all recruitment strategies used, on average (excluding personnel costs) the cost per participant randomized into the final sample was $49. Our ability to compare recruitment costs to other clinical trials among YACS is limited. However, our overall recruitment costs appear to be lower than other behavioral trials among young adults; for instance, recruitment of young adults into a weight gain prevention trial cost over $230 per participant randomized, with the most cost-effective method being email (~$38 per participant recruited) [54]. In a scoping review, social media was the most successful recruitment method in 4 of 11 intervention studies included [30] with one study identifying Facebook advertising as the most cost-effective recruitment strategy for recruiting participants to a trial of online intervention for anxiety and depression ($37 per participant) [55]. Overall, more studies are needed to understand the cost-effectiveness of different recruitment strategies to enroll YACS into clinical trials.

Our findings should be considered in the context of study limitations. Study participants may have been exposed to recruitment ads multiple times across different channels (e.g., Facebook post, booth at cancer conference), and we were unable to capture the potential overlap and multiple doses of recruitment exposures. As previously noted, our knowledge of the actual number of individuals approached through channels other than direct mail and the health registry was limited. We identified potentially eligible individuals using a hospital tumor registry local to the research site, so our findings may not generalize to other local and national tumor registries. Finally, we used recruitment strategies to varying degrees, depending on the earlier success of that strategy, so recruitment yields may vary accordingly. Despite these limitations, our study was strengthened by our formative work to guide recruitment messages. We made concerted efforts to work with community-based organizations serving YACS, which may have resulted in study posts from trusted organizations and sources. Additionally, our tracking of recruitment sources by capturing data from originating URLs enabled us to characterize yields and cost per participant across channels. The use of these approaches facilitated successful, efficient, and cost-effective recruitment of an underrepresented population into a behavioral clinical trial and may be useful for recruitment of YACS into cancer clinical trials more broadly.

## Conclusions

We recruited 280 YACS to a randomized trial of a remotely-delivered mHealth physical activity intervention at a rate of about 20 individuals per month. Unpaid social media, primarily through Facebook posts by organizations/friends, was the most successful recruitment strategy followed by direct mail to individuals identified through a local health registry. Social media posts were also the most cost-effective strategy, and the cost per participant (excluding personnel costs) across strategies was $49 per participant enrolled. Formative work was useful for guiding our recruitment messages and approaches, as was systematic tracking of recruitment yields by channel. These findings and approaches provide useful guidance for recruiting physically inactive YACS and for remotely-delivered intervention trials.

## Supplementary Information

**Additional file 1.** Recruitment messages evaluated in formative work.

**Additional file 2.** Organizations that shared IMPACT study recruitment information.

## Data Availability

The datasets generated and/or analyzed during the current study are not publicly available due to requirements to protect the privacy of participants, in accordance with their informed consent (University of North Carolina at Chapel Hill IRB Study #16-3409), but are available from the corresponding author on reasonable request.
