# Supplementary material for: Recruitment of young adult cancer survivors into a randomized controlled trial of an mHealth physical activity intervention
Source: Trials. 2022 Apr 4;23:254. doi: 10.1186/s13063-022-06148-5 (PMC8981777; doi:10.1186/s13063-022-06148-5)
Supplement: Supplementary file 1 — Additional file 1. Recruitment messages evaluated in formative work. [file 13063_2022_6148_MOESM1_ESM.pdf]

Article title: Recruitment of young adult cancer survivors into a randomized controlled trial of an mHealth physical activity intervention
Author names: Carmina G. Valle, Lindsey N. Camp, Molly Diamond, Brooke T. Nezami, Jessica Gokee LaRose, Bernardine M. Pinto, Deborah F. Tate
Corresponding author: Carmina G. Valle, PhD, MPH; University of North Carolina at Chapel Hill; carmina.valle@unc.edu

Additional file 1. Recruitment messages evaluated in formative work

---

Looking for ways to get back to your pre-cancer fitness levels on your time and at your convenience? Join our study to figure out how!

Looking for ways to get back to your pre-cancer physical activity levels on your time and at your convenience? Join our study to figure out how!

Are you struggling with fitness after cancer and interested in helping others with the same problem? Join our study to find out how you can help others like you!

Are you struggling with physical activity after cancer and interested in helping others with the same problem? Join our study to find out how you can help others like you!
