# Supplementary material for: Recruitment of young adult cancer survivors into a randomized controlled trial of an mHealth physical activity intervention
Source: Trials. 2022 Apr 4;23:254. doi: 10.1186/s13063-022-06148-5 (PMC8981777; doi:10.1186/s13063-022-06148-5)
Supplement: Supplementary file 2 — Additional file 2. Organizations that shared IMPACT study recruitment information. [file 13063_2022_6148_MOESM2_ESM.pdf]

Article title: Recruitment of young adult cancer survivors into a randomized controlled trial of an mHealth physical activity intervention
Author names: Carmina G. Valle, Lindsey N. Camp, Molly Diamond, Brooke T. Nezami, Jessica Gokee LaRose, Bernardine M. Pinto, Deborah F. Tate
Corresponding author: Carmina G. Valle, PhD, MPH; University of North Carolina at Chapel Hill; carmina.valle@unc.edu

Additional file 2. Organizations that shared IMPACT study recruitment information

15-40 Connection
A Ballsy Sense of Tumor
Cervivor
Hope Lab
Lacuna Loft
Northwestern University Adolescent & Young Adult Cancer Program
The SamFund
Stanford Adolescent and Young Adult Cancer Program
Stupid Cancer
Teen Cancer America
True North Treks
University of North Carolina at Chapel Hill Adolescent and Young Adult Cancer Program
